# Nearly Complete Genome Sequence of Brugia malayi Strain FR3

**DOI:** 10.1128/MRA.00154-20

**Published:** 2020-06-11

**Authors:** Alan Tracey, Jeremy M. Foster, Michael Paulini, Alexandra Grote, John Mattick, Yu-Chih Tsai, Matthew Chung, James A. Cotton, Tyson A. Clark, Adam Geber, Nancy Holroyd, Jonas Korlach, Silvia Libro, Sara Lustigman, Michelle L. Michalski, Matthew B. Rogers, Alan Twaddle, Julie C. Dunning Hotopp, Matthew Berriman, Elodie Ghedin

**Affiliations:** a Wellcome Sanger Institute, Wellcome Genome Campus, Hinxton, Cambridgeshire, United Kingdom; b Division of Protein Expression & Modification, New England Biolabs, Ipswich, Massachusetts, USA; c European Molecular Biology Laboratory, European Bioinformatics Institute (EMBL-EBI), Wellcome Genome Campus, Hinxton, Cambridgeshire, United Kingdom; d Department of Biology, Center for Genomics and Systems Biology, New York University, New York, New York, USA; e Institute for Genome Science, University of Maryland School of Medicine, Baltimore, Maryland, USA; f Pacific Biosciences, Menlo Park, California, USA; g Laboratory of Molecular Parasitology, Lindsley F. Kimball Research Institute, New York Blood Center, New York, New York, USA; h Department of Biology and Microbiology, University of Wisconsin Oshkosh, Oshkosh, Wisconsin, USA; i Department of Surgery, UPMC Children’s Hospital of Pittsburgh, Pittsburgh, Pennsylvania, USA; j Department of Microbiology and Immunology, University of Maryland School of Medicine, Baltimore, Maryland, USA; k Greenebaum Cancer Center, University of Maryland School of Medicine, Baltimore, Maryland, USA; l Department of Epidemiology, College of Global Public Health, New York University, New York, New York, USA; Broad Institute

## Abstract

Lymphatic filariasis affects ∼120 million people and can result in elephantiasis and hydrocele. Here, we report the nearly complete genome sequence of the best-studied causative agent of lymphatic filariasis, Brugia malayi. The assembly contains four autosomes, an X chromosome, and only eight gaps but lacks a contiguous sequence for the known Y chromosome.

## ANNOUNCEMENT

Brugia malayi is a causative agent of lymphatic filariasis, which affects ∼120 million people and can result in elephantiasis and hydrocele. An ∼71-Mbp B. malayi draft genome sequence was previously produced using Sanger sequencing with 8,180 scaffolds and an *N*_50_ value of ∼93 kbp (1, 2). Here, we used long-read sequencing and manually curated optical maps to complete this B. malayi genome.

B. malayi adult worms were obtained directly from the filarial nematode repositories at TRS Labs and the Filariasis Research Reagent Resource Center (FR3) (3)—the two major repositories that both independently maintain the same lineage of B. malayi originally from a green leaf monkey (4). High-molecular-weight genomic DNA was prepared by grinding frozen worms in liquid nitrogen and transferring them to 100 mM Tris-HCl (pH 8.5), 50 mM NaCl, 50 mM EDTA, 1% SDS, 1.1% β-mercaptoethanol, and 100 μg/ml NEB proteinase K at 55°C for 4 h with rocking. DNA was spooled from an ethanol precipitation following a phenol-chloroform extraction. DNA was suspended in Tris-EDTA (TE) (pH 8.0) with 25 μg/ml Epicentre RNase A at 37°C for 1 h followed by phenol-chloroform extraction, precipitation, and centrifugation at 12,000 × *g* at 4°C. Genomic DNA (30 μg) was sheared to 20 kbp with a Covaris g-TUBE. A 7-kbp Sage Science Blue Pippin size-selected SMRTbell library was prepared, and 11.3 Gbp of sequence data (3,922,808 reads; *N*_50_, 17,971 bp) were produced on a Pacific Biosciences RS II instrument (P5-C3; 180 min).

For optical mapping, individual phosphate-buffered saline (PBS)-washed B. malayi male worms were placed into ∼50-μl plugs of 1% InCert agarose (Lonza, Rockland, ME) in PBS that were extruded into 1 ml of 50°C 1% (wt/vol) *N*-lauroylsarcosine, 2 mg/ml proteinase K, and 0.5 M EDTA (pH 9.5) and incubated overnight with rocking at 50°C. Plugs were washed 5 times for 1 h each in TE (pH 8.0), with rocking at 4°C, and then stored at 4°C in 0.5 M EDTA (pH 8.0). Stretched and immobilized DNA was digested with NEB SpeI and AflII separately and fluorescently stained, generating ∼80× optical data depth. An OpGen Argus optical mapping system (2015 version), with proprietary MapManager (2015 version) and MapSolver version 3.1 software, resolved a 96.58-Mbp B. malayi SpeI optical map of 17 contigs and a 77.57-Mbp AflII optical map of 12 contigs.

The 1,895,591 PacBio subreads that passed a 0.75 quality filter (*N*_50_, 8,771 bp; mean, 5,930 bp) were assembled into 1,371 contigs with HGAP version 2 *de novo* assembly and compared to the *de novo* SpeI and AflII optical maps using MapSolver version 3.1. The genome was manually edited with publicly available capillary (2), Roche 454 (SRA accession number PRJNA10729), and Illumina (5) reads mapped to the PacBio contigs with Gap5 (6). Errors were corrected with three iterations of iCORN2 (7) with Bowtie mapping (8) using a tile path of 40× sequencing depth using pseudoreads created with the script to_perfect_reads (https://github.com/sanger-pathogens/Fastaq) using the prior publicly available WormBase assembly release 242 (WS242), followed by 3 further iterations using Illumina reads. Automated gap filling (24 iterations) was performed using IMAGE version 2.4.1 (9) and the Illumina reads. PBSuite_14.6.24 and smrtanalysis-2.2.0.133377, PBJelly (10), and Quiver were used to close gaps, add additional scaffolding, error correct, and trim. Introduced errors were corrected with three further iterations of iCORN using Illumina reads. Aligned sprai version 0.9.9.1-corrected (https://bioconda.github.io/recipes/sprai/README.html) PacBio reads were used for manual extension of sequence contigs, reducing the total gap count to 8. Default software parameters were used unless otherwise noted.

The resulting 87-Mbp assembly of 196 scaffolds has a GC content of 28% and an *N*_50_ value of 14.2 Mbp with 4 autosomes and an X chromosome but is lacking a contiguous Y chromosome despite numerous efforts to assemble it.

### Data availability.

This B. malayi v4 assembly with the WS270 annotation can be accessed at NCBI (accession number GCA_000002995.5), WormBase (http://www.wormbase.org/species/b_malayi), and WormBase-Para-Site (http://parasite.wormbase.org/Brugia_malayi_prjna10729/Info/Index/). The PacBio data are available at the SRA under accession number SRX3461807.
